# MCU Up‐regulation contributes to myocardial ischemia‐reperfusion Injury through calpain/OPA‐1–mediated mitochondrial fusion/mitophagy Inhibition

**DOI:** 10.1111/jcmm.14662

**Published:** 2019-09-09

**Authors:** Lichun Guan, Zhimei Che, Xiangdong Meng, Yong Yu, Minghui Li, Ziqin Yu, Hui Shi, Dicheng Yang, Min Yu

**Affiliations:** ^1^ Department of Cardiovascular Surgery Shanghai General Hospital, Shanghai Jiao Tong University, School of Medicine Shanghai China; ^2^ Department of Anesthesiology Renji Hospital, Shanghai Jiao Tong University, School of Medicine Shanghai China; ^3^ Department of Cardiology Shanghai Institute of Cardiovascular Diseases, Zhongshan Hospital, Fudan University Shanghai Shi China

**Keywords:** calpain, ischemia/reperfusion (I/R), mitochondrial calcium uniporter (MCU), mitochondrial fission, mitophagy

## Abstract

Mitochondrial dynamic disorder is involved in myocardial ischemia/reperfusion (I/R) injury. To explore the effect of mitochondrial calcium uniporter (MCU) on mitochondrial dynamic imbalance under I/R and its related signal pathways, a mouse myocardial I/R model and hypoxia/reoxygenation model of mouse cardiomyocytes were established. The expression of MCU during I/R increased and related to myocardial injury, enhancement of mitochondrial fission, inhibition of mitochondrial fusion and mitophagy. Suppressing MCU functions by Ru360 during I/R could reduce myocardial infarction area and cardiomyocyte apoptosis, alleviate mitochondrial fission and restore mitochondrial fusion and mitophagy. However, spermine administration, which could enhance MCU function, deteriorated the above‐mentioned myocardial cell injury and mitochondrial dynamic imbalanced. In addition, up‐regulation of MCU promoted the expression and activation of calpain‐1/2 and down‐regulated the expression of Optic atrophy type 1 (OPA1). Meantime, in transgenic mice (overexpression calpastatin, the endogenous inhibitor of calpain) I/R model and OPA1 knock‐down cultured cell. In I/R models of transgenic mice over‐expressing calpastatin, which is the endogenous inhibitor of calpain, and in H/R models with siOPA1 transfection, inhibition of calpains could enhance mitochondrial fusion and mitophagy, and inhibit excessive mitochondrion fission and apoptosis through OPA1. Therefore, we conclude that during I/R, MCU up‐regulation induces calpain activation, which down‐regulates OPA1, consequently leading to mitochondrial dynamic imbalance.

## 1 INTRODUCTION

Although revascularization is the most effective therapy to rescue ischaemic cardiomyocytes, reperfusion process could result in an extra cell loss and impair heart function. This phenomenon is known as ischemia/reperfusion injury (I/R).^1^ Mitochondria are enriched in cardiomyocyte and maintain their function by constantly undergoing fission and fusion and eliminating damaged part through mitophagy.^2^ Key proteins of mitochondrial dynamic pertain to GTPase proteins. Mitofusin 1 (MFN1), Mitofusin 2 (MFN2) and Optic atrophy type 1 (OPA1) located in mitochondrial membrane dominate mitochondrial fusion, while dynamin‐related protein1 (Drp1) translocates to the outer mitochondrial membrane and binds with receptors such as Fis1, MIEF, Mff, and mid49/51 to lead mitochondrial fission. PINK1 aggregates when the outer membrane potential of the damaged mitochondria decreases, attracts PARKIN to transfer to the outer mitochondrial membrane and initiates selective mitophagy. According to recent researches, the imbalance of mitochondrial fission, fusion and mitophagy plays vital role in I/R.^3^ I/R induces excessive mitochondrial fission and fragments, and down‐regulates fusion and mitophagy, which results in release of cytochrome C and caspase family proteins, and consequent apoptotic cascading effect.^4^ On the other hand, inhibition of mitochondrial fission or restoration of fusion and mitophagy seems protective in I/R.^5^

Mitochondrial calcium uniporter (MCU), localized in inner mitochondrial membrane (IMM), is the most important unidirectional channel responsible for Ca^2+^ influx into mitochondria. MCU regulates mitochondrial calcium homeostasis that is essential to ATP production and metabolism.^6^ When elevated cardiac output is demanded, MCU is a regulator of momentary mitochondrial Ca^2+^ loading to quickly match cardiac workload with ATP production.^7^ Nevertheless, upon cardiac I/R stress, MCU is responsible for mitochondrial Ca^2+^ overload, opening of the mitochondrial permeability transition pore (MPTP) and cell death.^8^ Down‐regulation of MCU by siRNA seemed protective from I/R injury in vitro.^8^ Recently, it was reported that the up‐regulation of MCU may even lead to increase intracytoplasmic calcium through sarcoplasmic reticulum‐mitochondria communication.^9^ However, whether MCU is involved in the defective mitochondrial fission/fusion and mitophagy in myocardial I/R injury, the underlying mechanism remains unknown.

Calpains belong to the calcium‐dependent thiol‐protease family and include 15 isoforms. Among them, μ‐calpain (calpain‐1) and m‐calpain (calpain‐2) were the main isoforms of calpains expressed in cardiomyocytes.^10^ During reperfusion, calpains are activated by calcium overload and play a role in I/R injury via cleavage of structural proteins and modification of pro‐apoptotic proteins.^11^ Calpain enhanced mitochondrial fission by activating calcineurin that phosphorylates the dynamin‐related protein 1 (Drp1) in neural cell model.^12^ However, if calpain was downstream regulated by MCU and impacted on mitochondrial fusion, the mitophagy during myocardial I/R is still elusive.

Located in IMM, OPA1 governs mitochondrial fusion.^13^ Disruption of OPA1 under pathologic conditions would lead to increase mitochondrial fission, fragmentation and even cell death.^14^ Recently, Zhang et al reported OPA1 down‐regulation associated with mitochondrial fusion and mitophagy inhibition in cardiac I/R model.^15^ Although OPA1 could be modulated by calpain in experimental neural cell,^16^ the relationship between calpain, OPA1 and MCU upon I/R injury remains unclear.

Therefore, this study investigated the role of MCU expression in I/R and its impact on mitochondrial fission, fusion and mitophagy via modulating calpain/OPA1 expression.

## 2 EXPERIMENTAL PROCEDURES

### 2.1 Animals

All animal experiments were approved by the Ethics Review Board for Animal Studies of Shanghai Jiao Tong University School of Medicine (approval No. SYKX‐2008‐0050; Shanghai, China) and were conducted in strict accordance with the guide for the care and use of laboratory animals published by the US National Institutes of Health (NIH Publication, 8th Edition, 2011). Adult male C57BL/6 mice (20‐30 g), purchased from Jackson Laboratory, were used in this study, and transgenic over‐calpastatin mice (Tg‐CAST, C57BL/6 background) were generously provided by Professor Ruizhen Chen (Zhongshan Hospital affiliated with Fudan University Heart Disease Institute, Shanghai, China). All mice were placed in a 12‐h/12‐h light/dark cycle and temperature‐controlled room with free access to tap water and food.

### 2.2 Ischemia/reperfusion model establishment

The process of I/R injury in mice was similar to the process of inducing myocardial infarction (MI).^17^ First, the mice (WT or tg‐cast) were fixed on the mouse plate, and 2% isoflurane gas was applied through the mask to anesthetize the mice.Then made a 1 cm incision in the left 4th intercostal space. After blunt dissection of the pectoralis major and sternal external muscles, the heart was gently squeezed out. The left coronary artery (LCA) was ligated with 6‐0 silk threads, and the heart was quickly pushed back into the chest. After 30 min's local ischemia duration, released the knot and achieved the reperfusion for 120 min(n=6/group). In sham operation groups, the chest was opened without LCA ligation (n=6/group).

Mitochondrial calcium uniporter inhibitor, Ru360, was dissolved in 0.9% saline solution and intraperitoneally administered (50nmol/kg) half an hour before surgery (n = 6/group). Ru360 inhibits MCU specially without affecting actomyosin ATPase activity, sarcolemmal Na^+^/Ca^2+^ exchange, sarcoplasmic reticulum Ca^2+^ uptake or release, L‐type Ca^2+^ channel current.

### 2.3 Infarct size estimation

After 30 minutes of ischemia and 120 minutes of reperfusion, LCA was re‐tied and 1% Evans blue solution (Sigma‐Aldrich) was injected into the right ventricle of the heart. The heart was quickly removed and frozen at −80°C, and was then cut into 2‐mm‐thick transverse slices. The slices were stained with 2% 2,3,5‐triphenyltetrazolium chloride (TTC) for 15 minutes. The myocardium was stained dark blue in the non‐ischaemic area. The area at risk (RA) was dyed red, while the infarct area (INA) was white. The INA and RA were captured by Stereomicroscope (Leica) and measured by ImageJ 1.37 software and expressed as the percentage of INA/R.

### 2.4 Morphological examination and apoptosis assays in risk area

Marginal area around I/R area was separated carefully from the heart. The tissue was fixed in 4% paraformaldehyde, desiccated, embedded in paraffin and sliced for TUNEL assay and immunohistochemistry. Apoptotic cardiomyocytes were detected by TUNEL assays with an in situ cell death detection kit (Roche) according to the kit protocol. DAPI was used to counterstain the nucleus. The images were obtained with an inverted fluorescence microscope (Nikon Eclipse TI‐SR). The cells in optionally selected areas were counted, and the apoptotic index (AI) was calculated as the number of TUNEL^+^ cell nuclei/total nuclei × 100%. After rehydration and microwave antigen retrieval, tissue slices are incubated with MCU antibodies (1:200, 14997S, CST) or calpain‐1 (1:200, 2539S, CST) or calpain‐2 (1:200, 2539S, CST) at 4°C overnight and incubated with secondary antibodies (K5007, Dako, Denmark) at 37°C for 30 minutes. The images were captured and analysed by Inversion Microscope (Leica Germany). For transmission electron microscope (TEM) analysis, cardiac tissue was fixed in 2.5% glutaraldehyde, dehydrated by gradient ethanol, embedded with pure acetone, sliced and stained with gold. TEM image was acquired by an electromicroscopic system (Leica Germany) (FEI Tecnai G2 Spirit).

### 2.5 Cardiomyocyte culture and induction of H/R

Neonatal rat (1‐3 days old, Department of Laboratory Animal Science, Fudan University) ventricular cardiomyocytes were isolated as previously described with a few modifications.^18^ In brief, hearts of neonatal mice were isolated surgically, and the ventricles were enzymatically digested in 0.1% collagenase II for 10 minutes in an automatic heat regulator at 37°C. After centrifugation and resuspension, cells were preplaced for 2 hours in Dulbecco's modified Eagle's medium (DMEM) supplemented with 10% foetal bovine serum (Gibco) to reduce fibroblasts, then plated in culture dishes and incubated at 37°C in a 5% CO_2_ incubator.

To mimic the I/R injury, myocytes were treated with hypoxia/reoxygenation (H/R). Briefly, the isolated myocardial cells, adhered to the well with normal pulsation, were moved to a hypoxia incubator (1% O_2_, 94% N_2_ and 5% CO_2_) for 12 hours, followed by reoxygenation in normoxic conditions for 3 hours at 37°C.

Calpain inhibitor I, PD150606, was supplemented to culture media half an hour before H/R to inhibit calpain activity with concentration of 10 μmol/L.

### 2.6 Cell viability and injury assay

Cell viability was measured by CCK8 assay, cardiomyocyte was incubated on a 96‐pore plate and pretreated (2000 cells/pore), and 10 µL CCK8 solution (Beyotime, Shanghai, China, C0037) was added to each pore. After incubation in cell incubator for 1 hour, the cardiomyocyte was detected by enzyme‐labelled instrument (Thermo, America) with a wavelength at 450 nm. The supernatant was collected after various treatments to measure the LDH levels using a commercial available kit (Beyotime Institute of Biotechnology, Shanghai, China; C0016) according to the manufacturer's instructions.

### 2.7 Western blot analysis

The marginal tissue of treated heart or cardiomyocytes was collected in an EP tube with RIPA protein lysis buffer containing protease inhibitors and phosphatase inhibitors, placed on ice for 30 min and then used to prepare tissue or cell protein. A BCA protein assay kit (Beyotime, Shanghai, China, Cat. No. P0010) was used to determine the protein concentration. The protein obtained above was separated by 12%‐15% SDS‐PAGE and then transferred onto a polyvinylidene difluoride (PVDF, Millipore, USA) membrane, placed in 5% BSA containing TBST at room temperature for 1h and then incubated overnight at 4°C with primary antibody diluted in TBST with 5% BSA. Primary antibodies include MCU, Drp1 (1:1000, 5361, CST), LC3 (1:1000, 4108, CST), calpain‐1, calpain‐2, OPA1 (1:1000, 80 471, CST), Bax (1:1000, 14 796, CST), Bcl‐2 (1:1000, 3498, CST), cleaved caspase‐3 (1:1000, 9664, CST), Pink1 (1:1000, 6946, CST) and Parkin (1:1000, 4211, CST). After that, PVDF membranes were washed with TBST three times and incubated with the HRP‐conjugated secondary antibody at room temperature for 1h. CoxIV (1:1000, 4850, CST) and GAPDH (1:10 000, 5174, CST) were used as internal control. An ECL‐HRP chemiluminescence kit was used to visualize the bands that were quantified by Image Lab Software (Bio‐Rad, USA). All samples were analysed in triplicate with a multilabel reader (excitation, 360 nm; emission, 460 nm, Thermo, America) and expressed as relative fluorescence units (RFU).

### 2.8 Calpain activity

A fluorometric calpain activity assay kit (ab65308, Abcam) was used to quantify calpain activity. In simple terms, the lysates of myocardial cells or heart tissue were centrifuged, and the supernatant was used to detect calpain activity using the fluorescent substrate N‐succinyl‐LLVY‐AMC. All samples were analysed in triplicate with a multilabel reader (excitation, 360 nm; emission, 460 nm, Thermo, America) and expressed as relative fluorescent units (RFU).

### 2.9 Confocal microscopy for the morphology of mitochondria

The cell culture medium was first removed, and MitoTracker Green (100 nmol/L, C1048, Beyotime) preheated to 37°C was added to wells. Cardiomyocytes with staining solution were incubated at 37°C for 30 minutes. After that, the staining solution replaced with fresh cell culture medium. These wells were observed under laser confocal microscopy Green (Leica Germany). The index of fragmentation is analysed by ImageJ. The mitochondrial length is measured by scale tools in Leica microscopy to present the level of mitochondrial fusion.

### 2.10 Flow cytometric detection of apoptosis

The Annexin V‐FITC Apoptosis Detection Kit microscopy (C1062, Beyotime Biotechnology) was applied under the manufacturer's instructions for apoptosis detection. In brief, primary cardiomyocytes were moulded, harvested after trypsinization treatments and washed twice with PBS; the cells were resuspended in 195 µL binding buffer, stained with Annexin V‐FITC and then subsequently incubated with both 5 µL of Annexin V‐FITC and 10 µL of the PI working substrate for 20 minutes at room temperature in the dark. Cellular fluorescence was measured by flow cytometry analysis (Becton‐Dickinson, USA). In the early stages of apoptosis, cells were Annexin V‐positive, while cells that were both PI‐ and Annexin V‐positive were at the later stages, whereas cells that were only PI‐positive were necrotic.

### 2.11 Cytoplasm and mitochondrial protein preparation

Cardiomyocytes were digested with Trypsin‐EDTA solution, centrifuged at room temperature (100‐200 *g*) and washed with PBS. Added mitochondria separation solution, the suspended cells were ground 20 times in the homogenizer and then centrifuged at 4°C (1000 *g*). The supernatant was carefully transferred to another EP tube and then centrifuged (10 000 *g*). Mitochondrial lysate (containing PMSF) was added into the precipitate (mitochondria) to obtain mitochondrial protein. The supernatant of the above steps continued to centrifuge (12 000 *g*), and cytoplasmic protein remained after abandoning the precipitate. The protein concentration was measured with the BCA protein assay kit. Unless otherwise specified, the above steps were experimented on ice.

### 2.12 Intracellular ATP detection

The mitochondria lysate liquid replaced the culture medium. After centrifugation (4°C, 5 min, 12 000 *g*), the supernatant was carefully aspirated and placed on ice. Next, 100 µL ATP working liquid was in each test well and laid aside for 3‐5 minutes at room temperature so that the background ATP was consumed. The sample above was added to the test wells in turn and rapidly mixed in a micropipette. Multiscan spectrum measures the wells in relative light units (RLU) by chemiluminescence (with a luminometer). Then, the ATP concentration was calculated according to the standard curve.

### 2.13 Fluorescence quantitative detection of the calcium ion concentration in mitochondria

Cell mitochondria Ca^2+^ concentration fluorescence quantitative detection reagent (GENMED SCIENTIFICS, GMS50097.1, USA), a type of mitochondrial calcium ion‐specific fluorescent probe (Rhod‐2), combined with calcium ions that can cause a remarkable increase in fluorescence intensity, was observed the relative change in the fluorescence peak under a fluorescence spectrophotometer instrument to determine the total calcium ion concentration in cellular mitochondria through classical technology and methods. A Mitochondrial Calcium Detection Kit was used for Ca^2+^ detection under the manufacturer's instructions. Briefly, mitochondria were harvested from cardiomyocytes for subsequent use. A black 96‐well cell culture plate was marked as bore cell samples, blank control (excluding mitochondria) and maximum contrast hole (excluding mitochondria). According to the instructions, reagents were added followed by incubation for 30 minutes at room temperature; then, incubation was performed for 30 minutes at 37°C under a fluorescence spectrophotometer instrument. All samples were analysed in triplicate with a multilabel reader (excitation, 550 nm; emission, 590 nm) and expressed in RFU.

### 2.14 RNA interference assays

According to the manufacturer's protocol, using interferon (Polyplus Transfection Hanbio Biotechnology Co. Ltd. China) for RNA interference, cardiomyocytes were transfected with 10 nmol/L of siRNAs against OPA1 purchased from Hanbio Biotechnology Co., Ltd. (China) and processed for 48h after transfection for controlling a non‐silencing RNA with scramble RNA.

OPA1 siRNA1.

Forward: CAGCAGUUCUCUUCUCUAA.

Reverse: UUAGAGAAGAGAACUGCUG.

Scramble siRNA.

Forward: UUCUCCGAACGUGUCACGU.

Reverse: ACGUGACACGUUCGGAGAA.

### 2.15 Statistical analysis

All statistical tests were analysed with GraphPad Prism 5, and the data were expressed as means ± SD. Statistical comparisons were conducted by Student's t test (for comparisons between two groups) or a one‐way analysis of variance (ANOVA, for comparisons of three or more groups) followed by the Bonferroni post hoc test. *P* < .05 was considered statistically significant.

## 3 RESULTS

### 3.1 MCU was up‐regulated under I/R injury, and MCU inhibition reduced infarction size and apoptosis in vivo

**Figure 1:**
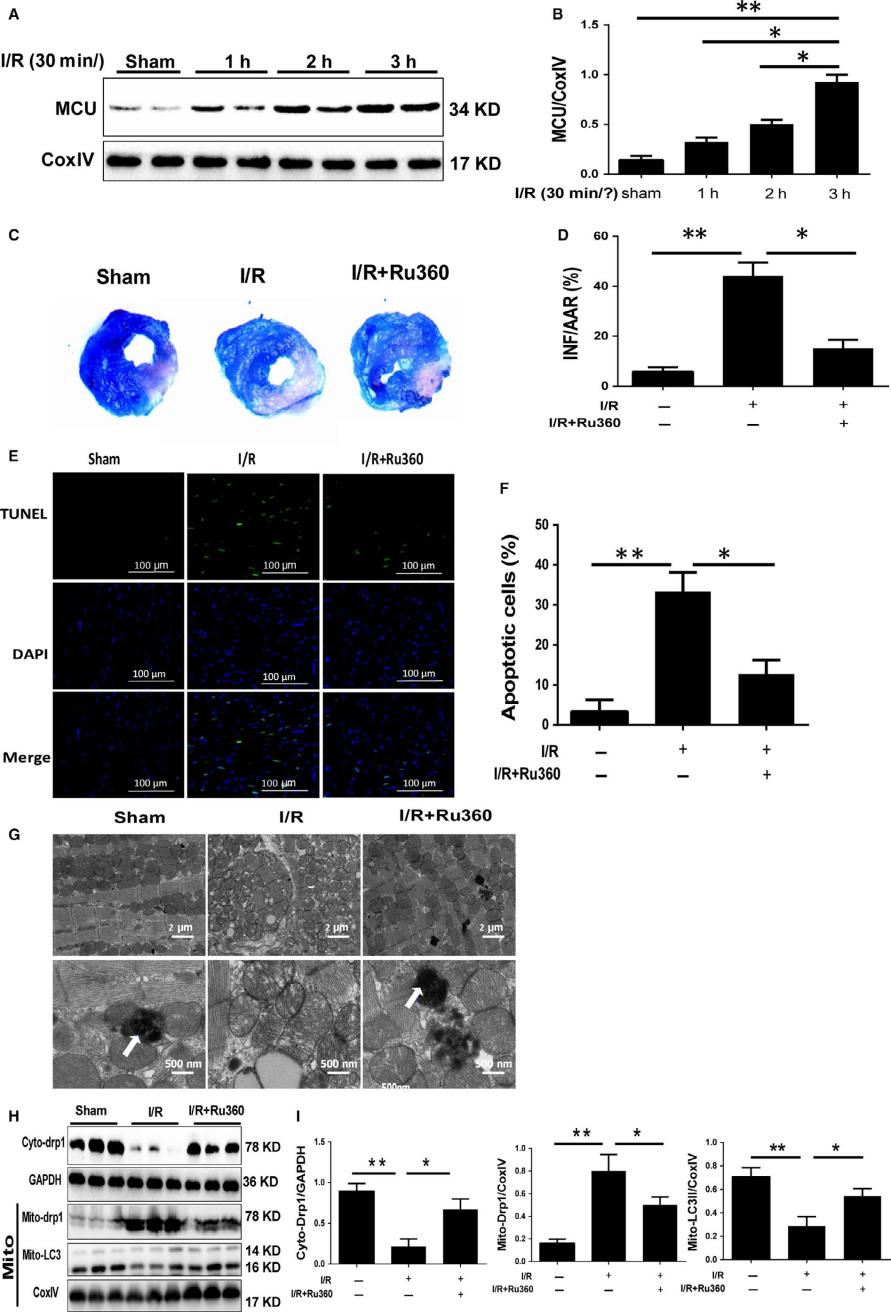
The expression of MCU during reperfusion, and the impact of Ru360 on myocardial infarction size and apoptosis after I/R injury. A and B, Western blot and quantitative analysis of MCU expression in cardiomyocytes of reperfusion area after 30‐min ischemia and 1‐h, 2‐h, 3‐h, 6‐h, and 12‐h reperfusion (I/R). C and D, Evans blue‐TTC staining showed the myocardial infarction size of sham, I/R surgery and Ru360 treatment plus I/R surgery. Quantification is shown. E and F, TUNEL staining showed levels of myocyte apoptosis of reperfusion area of three groups. Quantification is shown. G, Representative illustrations of TEM showing mitochondrial ultrastructural changes and autophagosome in each group. The scale bars were 2 μm and 500nm, respectively. Blue arrows represented autophagosome. H and I, Western blot showed expression of cyto‐Drp1, mito‐Drp1, mito‐LC3Ⅱ, respectively. Quantification is shown. Data are in accordance with normal distribution and expressed as means ± SD. N = 6 per group. *P < .05, **P < .01

After I/R surgery, MCU protein expression in reperfusion area was up‐regulated in a time‐dependent manner (Figure 1A,B). As MCU abundance was significantly increased after 3 hours of reperfusion and did not change afterword, we chose reperfusion for 3 hours in the subsequent experiments. To estimate the role of MCU on I/R injury, Ru360 was administrated to mice to inhibited MCU function. Infarction size and apoptosis were estimated by Evans blue‐TTC staining and TUNEL staining. Compare to sham group, I/R induced notable infarction and apoptosis in reperfusion area, while Ru360 significantly reduced infarction size (Figure 1C,D) and level of apoptosis (Figure 1E,F). These findings indicated I/R promoted MCU expression that was concerned with I/R injury.

### 3.2 MCU inhibition maintained mitochondrial morphology and homeostasis during I/R in vivo

Given the role of mitochondrial injury during I/R, we investigate the mitochondrial morphology by TEM. We found that the mitochondria were closely arranged with each other in a row, which was parallel to the sarcomere orientation in the sham group. In magnified image, the mitochondria were clubbed with dense and dark mitochondrial crista with no obvious vacuole formation, and autophagosomes could be detected. However, upon I/R the mitochondria were disordered, fragmented, swollen, and partly vacuolated and exhibited damaged crista in the I/R group. Meanwhile, the autophagosome failed to be scanned. Compared with the I/R group, those mitochondrial structure damages were mitigated with emergence of autophagosome in the I/R+Ru360 group (Figure 1G). Then, we estimated mitochondrial fission/fusion and mitophagy because these are essential for mitochondrial function. Cytoplasmic and mitochondrial Drp1 protein expression represents the level of mitochondrial fission, and mitochondrial LC3Ⅱ expression indicates the occurrence of mitophagy. Based on Western blot analysis, I/R‐induced Drp1 transferred from cytoplasm to mitochondria, indicating fission enhancement, and MCU inhibition alleviated Drp1 accumulation to mitochondria. Mito‐LC3Ⅱ decreased in I/R group; however, in I/R+Ru360 group, mito‐LC3 assembled significantly (Figure 1H,I). In summary, I/R resulted in damage of mitochondrial structure, increase in fission and decrement of mitophagy. MCU inhibition might benefit the balance of fission/fusion and mitophagy, and protected mitochondrial integrity.

### 3.3 MCU up‐regulation played a role in H/R‐induced myocardial injury in vitro

**Figure 2:**
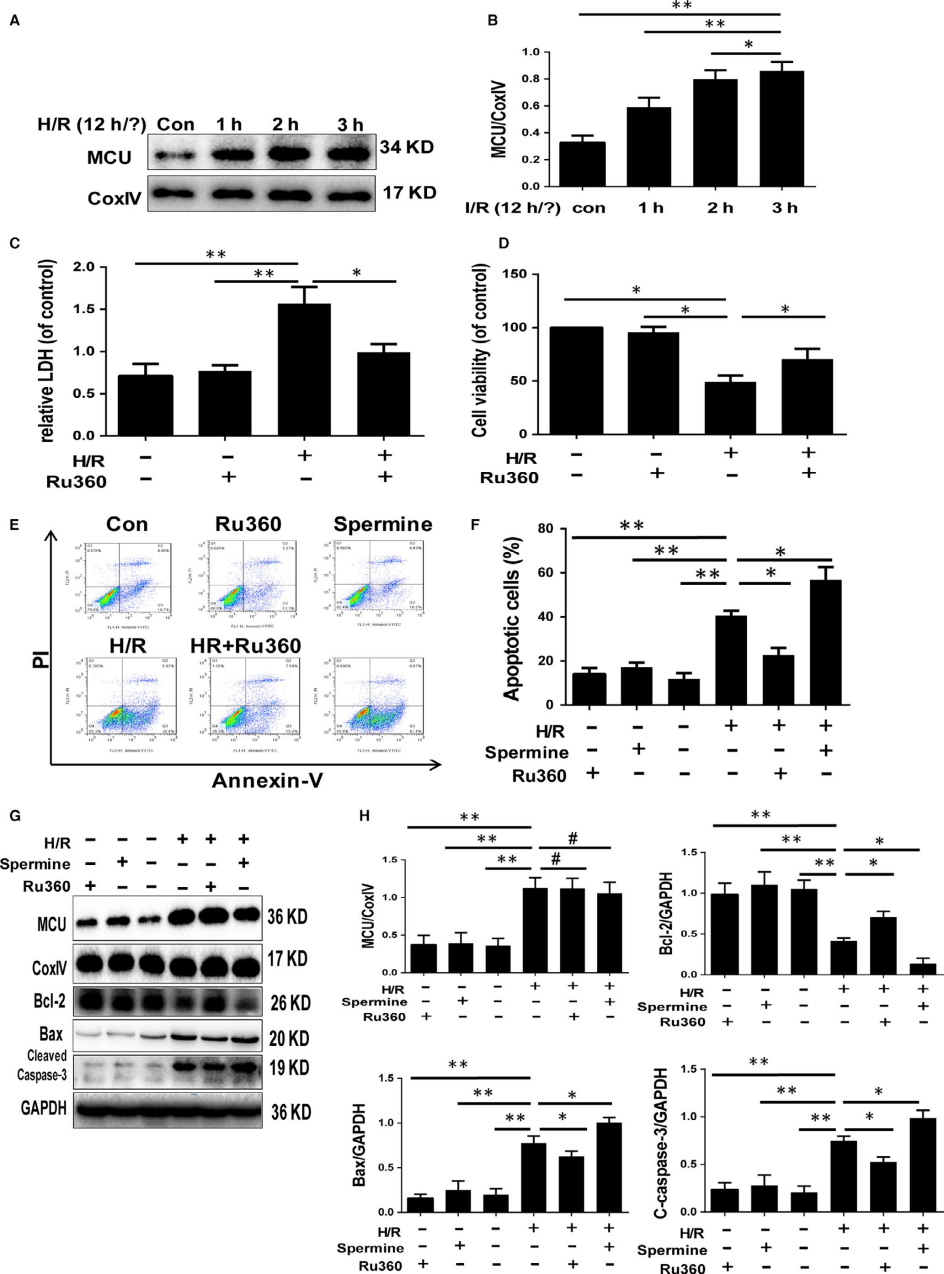
MCU expression after H/R, and the impact of MCU activation on cell injury and apoptosis. A and B, Western blot and quantitative analysis of MCU expression in primary cardiomyocytes after 12‐h hypoxia and 1‐h, 2‐h, 3‐h reoxygenation (H/R). C, Cellular viability by CCK8 assay. D, LDH release by ELISA. E and F, Cellular apoptosis by flow cytometry and quantitative analyses. G and H, Apoptotic protein expression by Western blot of primary cardiomyocytes and quantitative analyses after 12‐h/3‐h H/R, with or without Ru360/spermine treatment. Data are means ± SD from at least three different experiments. *P < .05, **P < .01

To confirm the effect of MCU on I/R injury and to clarify the role of MCU, a cultured neonatal mouse cardiomyocytes model with H/R treatment was used to mimic myocardial injury. As expected, MCU expression increased during reoxygenation course and rose the most significantly after 12‐h hypoxia and 3‐h reoxygenation (Figure 2A,B). Thus, we performed 12h/3h in H/R treatment protocol and introduced Ru360 as MCU inhibitor or spermine as MCU activator, which has no effect on myocytes in normal state (Figure 2C,D). The injuries of primary cardiomyocytes after H/R were estimated by CCK8 assay and LDH assay. H/R resulted in loss of viability and elevation of LDH release, which indicates myocyte injury. These injuries could be attenuated by Ru360 treatment or worsened by spermine (Figure 2C,D). Cellular apoptosis was measured using flow cytometry based on Annexin V‐FITC staining. H/R induced a high percentage of apoptosis, which could be significantly reduced by MCU inhibition and further increased with spermine (Figure 2E,F). Additionally, apoptosis was estimated by apoptosis‐related proteins including Bcl‐2, Bax and cleaved caspase‐3 by Western blot. In coincidence with flow cytometry results, H/R induced the increment of Bax and cleaved caspase‐3, and the decrement of Bcl‐2, and Ru360 dampened these apoptotic changes while spermine enhanced apoptosis (Figure 2G,H). These findings confirm that MCU was up‐regulated during I/R and contributed to I/R injury.

### 3.4 MCU activity modulated H/R‐induced mitochondrial fission/fusion, mitophagy and ATP production in vitro

**Figure 3:**
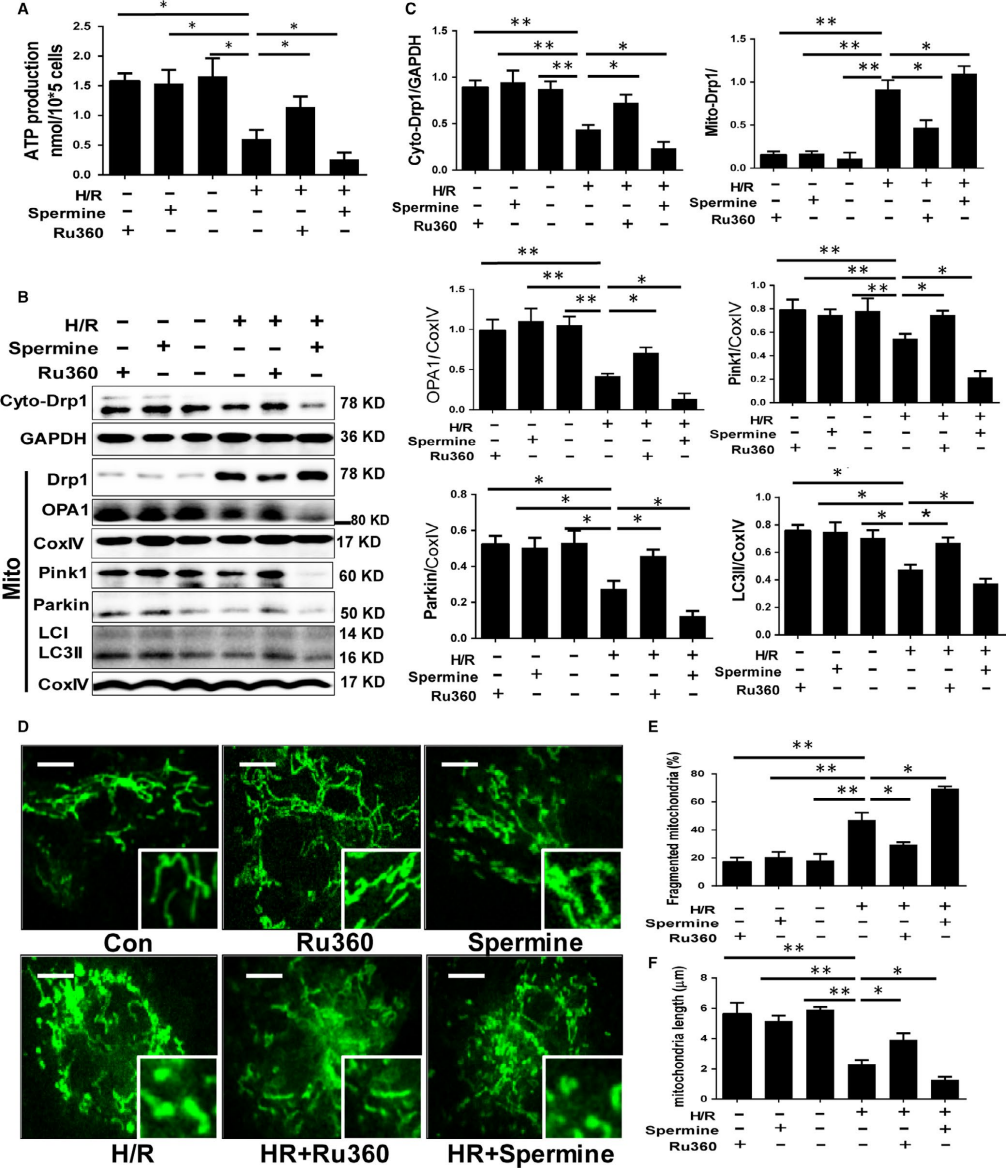
The impact of MCU inhibition and activation on ATP production, mitochondrial fission, fusion and mitophagy. A, ATP production. B and C, Protein expression of mitochondrial fission, fusion and mitophagy by Western blot assay and quantitative analysis. D‐F, Mitochondrial morphology by MitoTracker Green staining and confocal microscopy of primary cardiomyocytes and quantitative analyses after 12h/3h H/R, with or without Ru360/spermine treatment, and quantitative analysis. The scale of the mitochondrial images is 10µm. Data are means ± SD from at least three different experiments. *P < .05, **P < .01

To confirm the effect of MCU on mitochondrial function after H/R stimulation, we compared the cellular ATP production between the groups. The ATP production dropped markedly after H/R, while recovered with Ru360 treatment, and showed additional decrement with spermine treatment (Figure 3A). The key proteins of mitochondrial fission, fusion and mitophagy were examined by Western blot assay (Figure 3B,C). Drp1 transferred from cytoplasm to mitochondria after H/R, as well as OPA1, Parkin and LC3Ⅱ of mitochondria down‐regulated, indicating fission enhancement and fusion/mitophagy inhibition. These changes could be reversed by MCU inhibitor or be accelerated by MCU activator. Overwhelming fission and suppressed fusion/mitophagy could produce excessive mitochondrial fragments, which leads to apoptosis. We investigated the mitochondrial morphology by using MitoTracker Green staining and confocal microscopy (Figure 3D‐F). The mitochondrial network was presented as green points. The percentage of mitochondrial fragment rose in H/R group, while the average length of mitochondria dropped, indicating accumulation of mitochondrial fragment. Inhibiting MCU by Ru360 could dampen the fragmentation and restore the elongation, while activating MCU by spermine motivated the effect of H/R. These data clarified that MCU up‐regulation damaged the mitochondrial function, promoted fission, inhibited fusion and down‐regulated mitophagy, and thus produced excessive fragments, which was accordant with the aforementioned findings of apoptosis.

### 3.5 MCU modulated calpains expression and activity under I/R stress in vivo and in vitro

**Figure 4:**
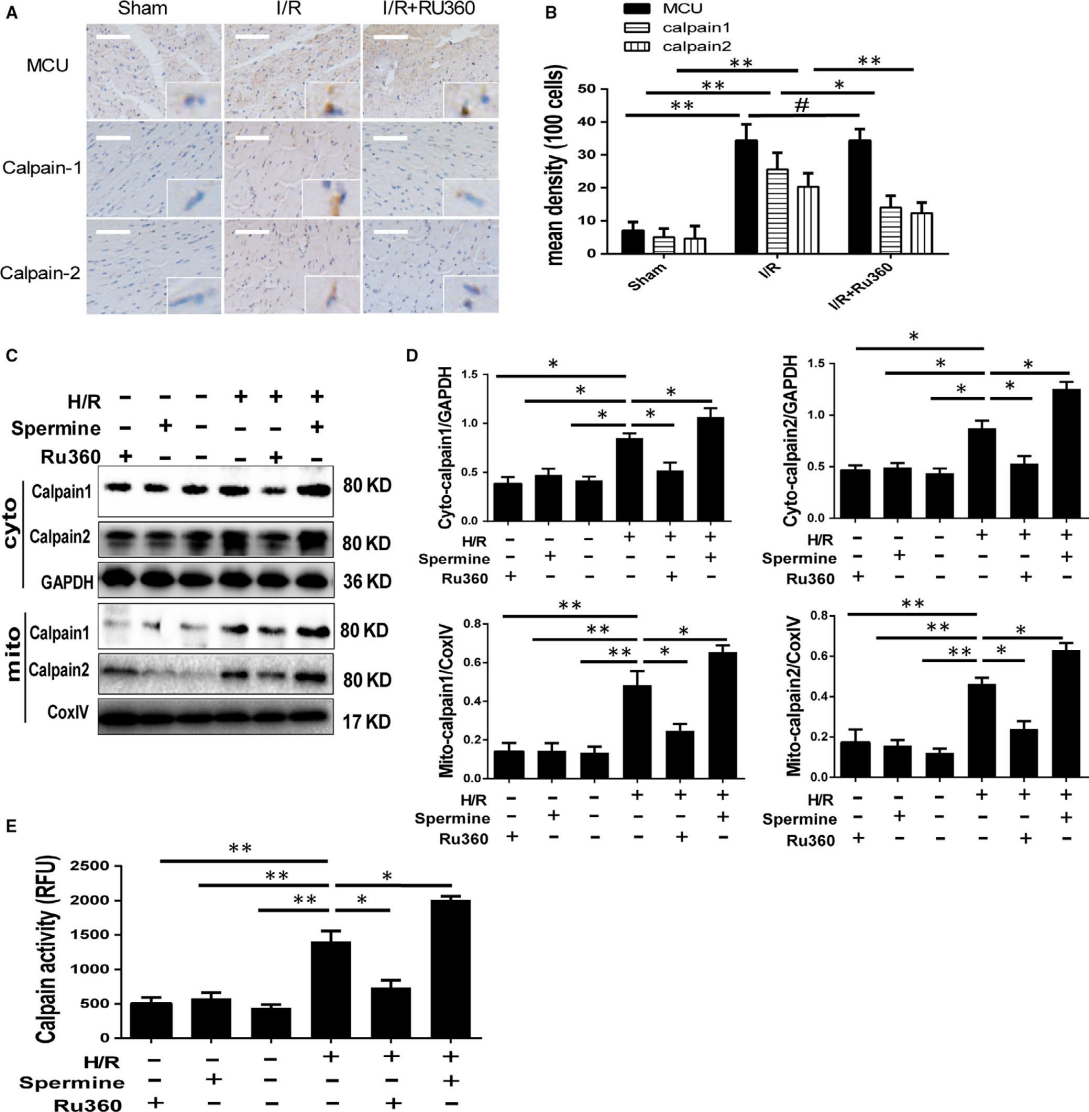
Expression and activity of calpain‐1 and calpain‐2 under I/R treatment and Ru360/spermine intervene. A and B Calpain‐1, calpain‐2 and MCU expression by immunohistochemical stains in reperfusion myocardium under experimental I/R injury and quantitative analyses. The magnification is 100 and 400 times. C and D Calpain‐1 and calpain‐2 expressions, respectively, in cytoplasm and mitochondria in primary cardiomyocytes by Western blot under H/R stimulation and Ru360/spermine intervene, and quantitative analyses. E Calpain activity by fluorescence quantitative analyses. Data are means ± SD from at least three different experiments. *P < .05, **P < .01

To further explore the downstream of MCU modulation, we investigated the expression and activity of calpains, which is a type of calcium‐depended protease after I/R injury in vivo and in vitro. Based on histopathology and immunohistochemical stains, the expression of MCU, calpain‐1 and calpain‐2 was detected in slices of myocardial tissue of I/R area (Figure 4A,B). After I/R surgery, the positive area of MCU, calpain‐1/2 rose correspondingly. Although MCU expression maintained with Ru360 treatment, calpain‐1/2 expression was down‐regulated. Consistent with the data in vivo, calpain‐1 and calpain‐2 expressions were increased equally in cytoplasm and mitochondria in primary cardiomyocytes subjected to H/R injury and were accompanied by the increment of calpain activity (Figure 4C‐E). Inhibiting MCU could blunt such effects, while activating MCU showed the opposite effects (Figure 4C‐E). In the molecular level, cellular free calcium concentration was varied in synchronicity with the expression of calpains (Figure S1A). Taken together, these evidence suggested MCU modulated calpains expression via cellular calcium homeostasis.

### 3.6 Tg‐CAST mice showed less myocardial injury, reduced mitochondrial fission and improved mitochondrial fusion an mitophagy during I/R by calpain inhibition

**Figure 5:**
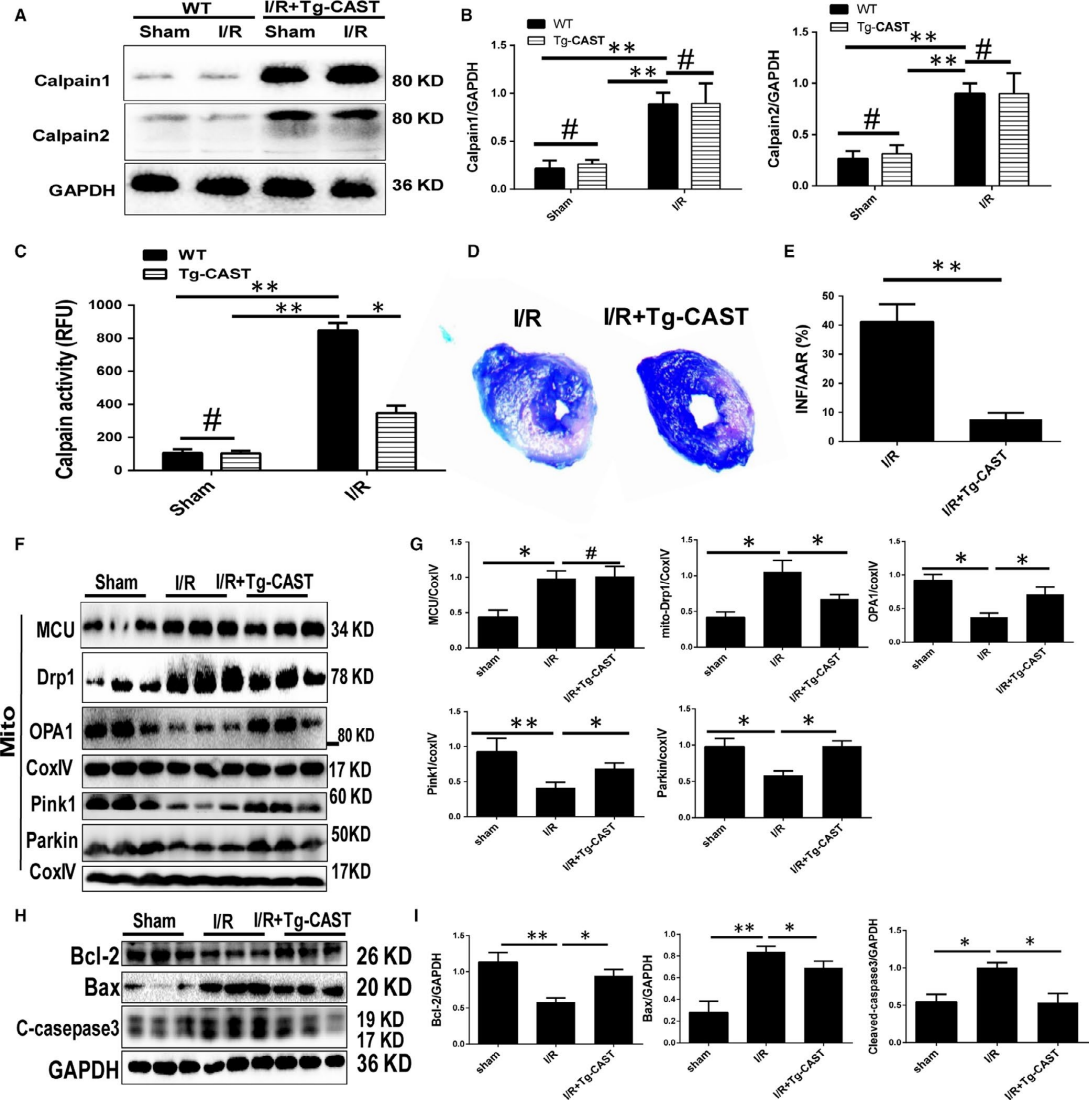
Impact of calpain inhibition on I/R injury and mitochondrial homeostasis in Tg‐CAST mice. A‐C, Calpain expression and activity in WT and Tg‐CAST mice under I/R surgery, and quantitative analyses. D and E, Myocardial infarction size by Evans blue‐TTC staining in WT and Tg‐CAST mice under I/R surgery, and quantitative analyses. F and G, Expression of MCU and proteins represents mitochondrial fission, fusion and mitophagy. H and I Expression of proteins represents apoptosis by Western blot in WT and Tg‐CAST mice under I/R surgery, and quantitative analyses. Data are in accordance with normal distribution and expressed as means ± SD. N = 6 per group. *P < .05, **P < .01

To further confirm the role of calpain activation in MCU‐mediated I/R injury and mitochondrial disorder, Tg‐CAST mice were used because calpastatin is a specific endogenous calpain‐1/2 inhibitor without interfering other proteins. Firstly, the transgenic mice were genotyped to identify the correct littermates (Figure S1B). Then, calpain protein expression and activity were examined. In normal state, the WT and Tg‐CAST mice showed similar calpain protein expression. After I/R surgery, though the expression of calpain protein was elevated equally in both groups, calpain activity was significantly lower in Tg‐CAST group than in WT (Figure 5A‐C). Myocardial infarction size of Tg‐CAST mice was reduced after I/R surgery compared to WT mice (Figure 5D,E). Based on Western blot assay, we found the up‐regulation of MCU was not affected by calpain inhibition in Tg‐CAST mice under I/R injury, indicating MCU is the upper stream of calpains. But calpain inhibition took effect on mitochondrial fission, fusion and mitophagy. I/R induced up‐regulation of mitochondrial Drp1 expression and down‐regulation of mitochondrial OPA1, PINK1 and Parkin expression, while calpain inhibition neutralized these changes in Tg‐CAST mice (Figure 5F,G). Additionally, I/R induces apoptosis that represented as increment of cleaved caspase‐3 and Bax, and decrement of anti‐apoptotic factor Bcl‐2. Calpain inhibition in Tg‐CAST mice under I/R recovered the balance between Bax and Bcl‐2, and reduced c‐caspase‐3 (Figure 5H,I). Overly, these data implied calpain up‐regulation and activation plays a role in I/R injury and MCU induced excessive mitochondrial fission and suppressed fusion/mitophagy.

### 3.7 The protective effect of calpain inhibition on mitochondrial fission/fusion and mitophagy could be abolished by OPA1 knockout

**Figure 6:**
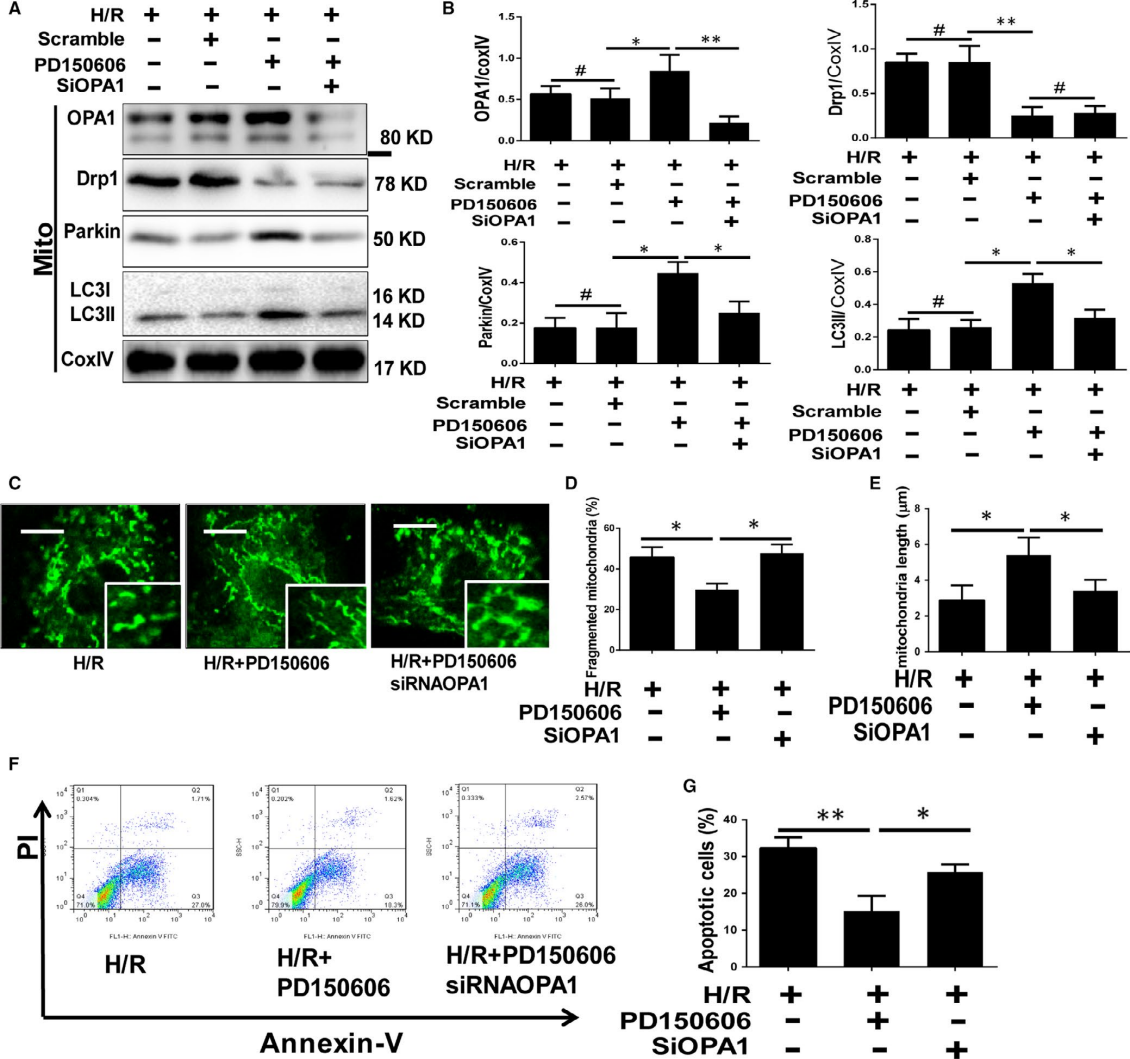
The impact of OPA1 knockout on mitochondria injury and apoptosis in vitro. A and B, Western blot assay of mitochondrial fission‐, fusion‐ and mitophagy‐related proteins in H/R myocytes with PD150606 and OPA1 siRNA pretreatment in vitro and quantitative analyses. C‐E, Mitochondrial morphology was observed using MitoTracker Green staining, plus the index of fragmentation and length was analysed. The scale of the mitochondrial images is 10 µm. F and G, Cellular apoptosis was detected by flow cytometry in H/R myocytes with PD150606 and OPA1 siRNA pretreatment in vitro and quantitative analysis. Data are means ± SD from at least three different experiments. *P < .05, **P < .01

To investigate the role of OPA1 in MCU‐calpain–mediated I/R injury and mitochondrial disorder, we introduced PD150606 (10 μmol/L) as calpain inhibitor, and OPA1 siRNA to down‐regulate OPA1 in primary cardiomyocytes before H/R was performed. The efficiency of OPA1 siRNA was verified by Western blot, and the expression of OPA1 was significantly reduced after siRNA transfection (Figure S1C). Mitochondrial OPA1 expression was reduced during I/R and recovered when calpain or MCU was inhibited (Figures 3B and 5F). Mitochondrial proteins were examined in myocytes under H/R with PD150606 and OPA1 siRNA pretreatment (Figure 6A,B). Consistent with the data in vivo, calpain inhibition abolished the exorbitant Drp1 accumulation, with recovered OPA1, LC3Ⅱ and Parkin. However, OPA1 knockout nullified the protective effect of PD150606 on OPA1, LC3Ⅱ and Parkin, except Drp1. Based on fluorescent imaging, mitochondrial network showed the fission and fusion using the percentage of fragment and length (Figure 6C‐E). As expected, calpain inhibition normalized the balance between fission and fusion, and OPA1 knockout increased the fragmentation and decreased the length again. In accordance with the changes in mitochondrial morphology, PD150606 pretreatment protected the H/R myocytes from apoptosis (Figure 6F,G), and OPA1 deletion rose the percentage of apoptosis again. These data suggested that the role of OPA1 in the MCU‐calpain mediated mitochondrial fusion/mitophagy inhibition, which results in mitochondrial apoptotic fission.

## 4 DISCUSSION

In this study, we demonstrated one of the mechanisms of myocardial I/R injury. MCU is up‐regulated during myocardial I/R, which increases the expression of cytoplasm and calpains and activates them. Activated calpains down‐regulate OPA1‐mediated mitochondrial fusion and mitophagy, promote mitochondrial fission, damage mitochondrial homeostasis and mitochondrial function, and promote apoptosis.

Several studies have shown that MCU is over‐open during I/R and participates in I/R injury through the mechanism of MCU‐Ca^2+^overload‐mPTP opening.^8^, ^19^, ^20^ In the present study, mitochondrial morphology was observed by TEM, and I/R could cause mitochondrial swelling and crista morphological disorder. At the same time, ATP production decreased, and myocardial injury and apoptotic expression increased. Consistent with the literature, Ru360 could protect mitochondrial morphology, partially restore ATP production, reduce infarct size and cell apoptosis when inhibiting MCU opening. MCU can also impact mitochondrial dynamics through several signalling pathways. In the I/R model of hippocampus cells, MCU up‐regulated the expression of mitochondrial fission–related protein Drp1/Fis1/MIEF and promoted mitochondrial fission.^21^ In pressure overload‐induced heart failure model, MCU is up‐regulated and inhibited mitophagy.^22^ In this study, through the intervention of inhibitor Ru360 and agonist spermine on MCU function, we demonstrated that MCU promoted Drp1 to migrate to mitochondria; down‐regulated OPA1, PARKIN and mito‐LC3Ⅱ; and played a role in promoting mitochondrial fission, inhibiting mitochondrial fusion and mitophagy. Mitochondrial network morphology is characterized by increased fragmentation and decreased mitochondrial length. Mitochondrial fragmentation eventually leads to endogenous apoptosis.

To further explore how MCU regulates the signalling pathway of mitochondrial dynamics, we found that the expression and activity of calpains in mitochondria and cytoplasm are regulated by MCU, while calpain regulates the key protein OPA1 of mitochondrial fusion. By observing the downstream effects of MCU inhibition and activation, we can infer that the up‐regulation and activation of calpain expression in mitochondria during I/R is the result of the high expression of MCU and the large amount of Ca^2+^ entering mitochondria. On the other hand, MCU may affect the concentration of calcium in cytoplasm through the interaction between mitochondria and endoplasmic reticulum to reach the result of calpains in cytoplasm.^9^ Previous studies have shown that calpain activation can cause cardiac dysfunction and apoptosis through a variety of signalling pathways.^23^ In mitochondria, activated calpain cleaves and modifies apoptosis‐inducing factor in mitochondria to release it from mitochondria to cytoplasm and initiate apoptosis.^24^ Recently, Ni R et al reported that calpain can degrade mitochondrial respiratory chain protein ATP synthase‐a (ATP5A1), interfere with ATP synthesis and promote ROS production.^25^ In this study, Tg‐CAST model was established to observe the effect of calpain on mitochondrial dynamics. We found that calpain activation promotes mitochondrial fission, inhibits fusion and mitophagy and aggravates apoptosis. At present, the effect of calpain on mitochondrial fusion‐related protein has been rarely reported. Calpain can inhibit the acetylation of MFN2 in liver I/R injury, which results in the interference of mitophagy and the damage of mitochondrial function.^26^ In the model of neuroblastoma SH‐SY5Y cells and cerebellar granule neurons, calpain activation down‐regulates OPA1, inhibits mitochondrial fusion and increases mitochondrial fragmentation.^12^, ^16^ We speculate that calpain affects mitochondrial fission/fusion and mitophagy by regulating OPA1, but it is not clear whether calpain has direct or indirect effects on OPA1.

In myocardial injury I/R, promoting mitochondrial fusion and autophagy can improve mitochondrial morphology, increase ATP production and reduce mitochondrial‐derived apoptosis.^27^ In the retinal ganglion cells model, OPA1 dominant not only mitochondrial fusion, but also mitophagy.^28^ In recent years, increasing evidence has shown that OPA1 has protective effects in various cardiac diseases, especially in I/R.^29^ Elevating the expression of OPA1 by drug preconditioning or overexpression of OPA1 in transgenic cells could improve the morphology and function of mitochondria and reduce myocardial injury.^30^, ^31^ However, conditioned OPA1 knock‐down aggravates mitochondrial dysfunction and cell damage.^32^ This study found that OPA1 is downstream of MCU/calpain. Up‐regulation of MCU/calpain inhibits OPA1 during I/R, inhibits mitochondrial fusion and autophagy, promotes mitochondrial fragmentation and initiates apoptotic process.

In conclusion, we established I/R models in vivo and in vitro and demonstrated that MCU is up‐regulated during I/R and activates calpain by mitochondrial calcium overload. And then, calpain inhibits OPA1; thus, mitochondrial fusion and mitophagy are inhibited to promote mitochondrial fission, leading to mitochondrial morphological and functional damage, and activation of intrinsic apoptosis pathway. This study suggests that MCU‐calpain‐OPA1 signalling pathway is, at least, one of the mechanisms of myocardial I/R injury (Figure S1D）.

## CONFLICTS OF INTEREST

The authors declare that they have no conflicts of interest.

## AUTHORS’ CONTRIBUTION

The study was designed by Min Yu, Dicheng Yang and Lichun Guan. The animal model was set up by Lichun Guan and Zhimei Che. The molecular biological experiment such as PCR, Wester blots, flowmetry study and immunofluorescence staining; ATP and calcium ion detection were carried out by Lichun Guan, Zhimei Che, Yong Yu, Xiangdong Meng and Ziqin Yu. Cell culture and virus transfection were done by Lichun Guan and Zhimei Che. Morphological study such as TEM, TTC staining, morphological examination and Evans blue‐TTC staining was implemented by Lichun Guan and Yong yu. Finally, the statistical analysis and article writing were done by Min Yu, Lichun Guan and Dicheng Yang.
