# Supplementary figures and images for: MCU Up‐regulation contributes to myocardial ischemia‐reperfusion Injury through calpain/OPA‐1–mediated mitochondrial fusion/mitophagy Inhibition
Source: J Cell Mol Med. 2019 Sep 9;23(11):7830–43. doi: 10.1111/jcmm.14662 (PMC6815825; doi:10.1111/jcmm.14662)

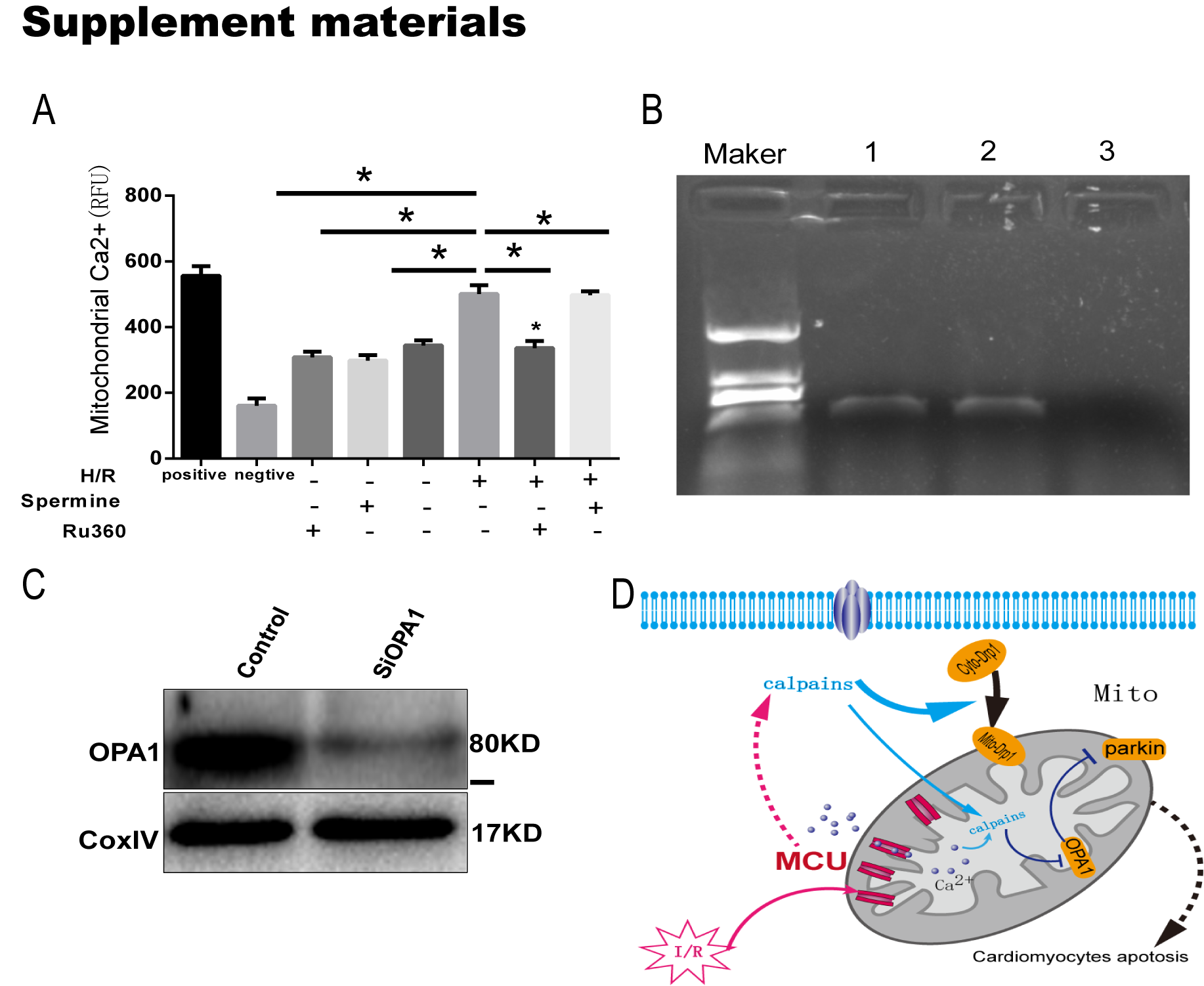

Supplement: Supplementary file 1 [file JCMM-23-7830-s001.tif]
